# Does Emotional Intelligence Increase Satisfaction with Life during COVID-19? The Mediating Role of Depression

**DOI:** 10.3390/healthcare9111529

**Published:** 2021-11-09

**Authors:** Orhan Koçak

**Affiliations:** Department of Social Work, Faculty of Health Sciences, Istanbul University-Cerrahpasa, Istanbul 34320, Turkey; orhan.kocak@istanbul.edu.tr

**Keywords:** emotional intelligence, COVID-19, depression, satisfaction with life

## Abstract

COVID-19 has spread rapidly and become a health crisis around the world, and negatively affected the mental state of individuals. Emotional intelligence (EI) can play an important role in coping with the mental problems experienced due to the pandemic. This study examined how individuals’ emotional intelligence levels affect depression and satisfaction with life during the COVID-19 period. The study was designed as quantitative and cross-sectional and reached 578 adult participants online. Emotional intelligence trait scale–short form, depression subscale (DASS-21), satisfaction with life scale, and sociodemographic questions as control variables were used as data collection tools in the study. The data obtained were conducted using SPSS 24, PROCESS-Macro, and Amos 25 statistical programs. The hypotheses established were tested by correlation, multiple regression, mediating, and moderating analyzes. Results confirmed that emotional intelligence had a positive association with satisfaction with life and a negative association with depression. In addition, interaction analyses found that age and family type had a moderating effect on satisfaction with life, and depression had a mediating effect. After discussing the importance of emotional intelligence as a coping mechanism in dealing with problems, some suggestions were made to policymakers and practitioners.

## 1. Introduction

The new type of coronavirus disease, which was first identified in Wuhan, China, in December 2019, was defined on 7 January 2020, and the first COVID-19 case in Turkey was detected on 11 March 2020. COVID-19 has affected the whole world in a short time due to its rapid spread and severe clinical course. Furthermore, it has been observed that the severity and intensity of negative emotions caused by the uncertainty about the course of the disease and when it will end cause depressive effects in different ways from person to person [1]. This difference is thought to be related to the level of emotional intelligence [2,3,4,5,6,7]. Emotional intelligence is the ability to understand, evaluate, direct and manage emotions [8,9]. Understanding and regulating emotions also positively affect coping skills with problems, problem-solving, controlling negative emotions, and in decreasing psychological outcomes such as depression. Depression is a mental illness that affects over 300 million individuals worldwide [10]. It is characterized by constant sorrow and a loss of interest and pleasure in enjoyable activities for the individual. The most typical symptoms are stagnation, intense sadness, and a slowdown of thought, speech, movement, and physiological functions. Furthermore, hopelessness, worthlessness, helplessness, sadness, hesitation, inability to act, incapacity to continue working, and feelings of guilt are significant signs. Therefore, decreasing depression will positively trigger satisfaction with life which is related to comparing the individual’s life expectancy with the result obtained, the positive emotions they feel about their lives, and the degree of satisfaction with life [11,12]. 

The problems caused by the COVID-19 virus are multifaceted. While the resulting problems cause health, economic, social, and psychological problems, these problems also trigger each other. For example, factors such as unemployment, isolation, and fear, stress and anxiety about illness in this period negatively affect the psychology of individuals. Therefore, the COVID-19 pandemic should be examined not only with its physical but also with its spiritual, psychological, social, economic, and political consequences, and measures should be taken accordingly [13]. In this regard, emotional intelligence’s protective effect is critical in alleviating rising individual anxieties and psychological problems during the COVID-19 era [14,15,16]. Individuals with high emotional intelligence are expected to have a lower depressive affect and higher life satisfaction. 

This study was based on the theory of emotional intelligence. The emotional intelligence theory suggests that individuals who are skilled at reflecting, understanding, and regulating emotions will have better psychological and social well-being [17]. A research question was asked in this study RQ: How do emotional intelligence and sociodemographic factors have a role in increasing satisfaction with life? Emotional intelligence was found to increase satisfaction with life, and depression had a mediating role in this effect. It was understood that satisfaction with life increases with rising socioeconomic status (SES), decreases in advanced ages, and was more in women. Emotional intelligence, which effectively reduces depression, is an essential trait in dealing with extraordinary conditions such as pandemics.

## 2. Theoretical Background of the Research Hypotheses

### 2.1. Emotional Intelligence as Research Theory

Researchers have studied the concept of intelligence and emotions for many years. Certain emotions may support certain types of thinking. In this sense, positive emotions can generally lead to better decision-making and increase productivity [18,19]. The concept of emotional intelligence was introduced to the literature by Mayer [20,21]. Both intelligence and emotion are active in all mental processes. In a way, emotion and intelligence are in a mutual relationship in mental activities [22]. Emotional intelligence has been defined as a type of intelligence that includes the ability of individuals to observe and understand their own and others’ emotions and to use their emotions as a guide to their behavior [23,24]. To be qualified as emotionally intelligent, individuals must be skilled in defining, understanding, using, and regulating their emotions [25,26]. Goleman defines emotional intelligence as being able to take action, move on despite problems, control impulses, regulate mood, control negative thoughts, empathize, and hope [27]. Bar-on considered emotional intelligence as non-cognitive intelligence and described the term (EI) as a set of emotional, personal, and interpersonal talents, skills, and abilities that influence one’s capacity to deal with daily challenges [28]. Emotional intelligence is regarded as the whole of interrelated emotional competencies, skills, and facilitators that determine how much we understand and express ourselves, how we understand and relate to others, and our capacity to cope with problems [29].

Emotional intelligence enables individuals to understand and evaluate their own and other people’s emotions and use information about emotions effectively in daily and professional life. Accordingly, persons can be classified as “emotionally intelligent” if they can control their emotions as they like and accomplish the required results in their professional or private lives [30,31]. Thanks to emotional intelligence, the development of individuals’ thoughts are ensured by reasoning about emotions [17]. Hence, the basic assumption under the concept of emotional intelligence is the capability to take into account, organize and express emotions and analyze the emotions of others correctly. Furthermore, a person’s resilience in the face of rapid changes and problems and the ability to cope with them. Emotional intelligence enables people to deal with adversity such as COVİD-19 pandemic conditions by strengthening their decision-making abilities, communication skills, and psychological resilience [16]. It serves as a basis for developing feelings in a healthy manner, allowing individuals to manage different circumstances effectively and thus also helps relieve post-traumatic stress disorders (PTSD) [14,15,32].

### 2.2. Emotional Intelligence and Depression

The literature reveals that emotional intelligence is a coping trait for physical and mental disorders such as depression, anxiety, and stress [33,34,35,36,37]. Depression is a disease that is very common among people, may differ with age, and is characterized by changing symptoms [38,39]. It has a symptom cluster that can occur in a wide range, from normal sadness to severe psychotic symptoms [40]. Individuals suffering from this condition have difficulty performing normal daily activities. Their social and occupational functionality is impaired, appetite deterioration and sleep problems are observed, attention may decrease, and slowness in thinking may be experienced. In addition, symptoms such as self-harming behaviors, suicide attempts, reduced libido, pessimism, guilt, and decreased self-confidence can be seen. There are some social, economic, emotional, and biological reasons for the emergence of depression. For example, age, gender, race, marital status, social environment, socioeconomic level, negative life experiences, loss of a loved one, family problems, and physical problems affect depression levels in individuals [41].

Emotional intelligence empowers the fight against many individual and social problems. Emotional intelligence strengthens the fight against addiction by increasing individuals’ emotion and problem-focused coping skills [42]. Similarly, studies in the literature show that there is a relationship between emotional intelligence and depression. Many studies have found that emotional intelligence reduces depression [43,44,45,46]. A study found that students who could not physically go to school during the COVID-19 period in India used their emotional intelligence capacity to cope with the educational problems they experienced [47]. Since EI is higher in women than in men, an increase in EI does not cause a decrease in depression in women as much as in men [43]. While a study found a negative relationship between emotional intelligence and depression, the moderator effect of gender was found in the relationship. While low emotional intelligence led to higher depression in men, the same result did not occur in women [48]. A high level of emotional intelligence was connected to reduced levels of anxiety and depression and improved social and physical health levels in studies of university students and adolescents in Spain [49,50]. Emotional intelligence may reduce not only depression but also suicidal thoughts and behaviors. A study conducted among nursing students found a negative relationship between emotional intelligence and suicidal ideation and suicide risk, which emerge with depression and stress [51]. Another study conducted with nursing students found that emotional intelligence minimized the students’ stress levels, and thus their perceived social support and mental health status were better [52]. There are similar results in many studies in the literature [53,54,55]. A study conducted with the elderly in the USA observed that as emotional intelligence increased, the risk of being exposed to depression decreased. Therefore, the rising emotional intelligence made a beneficial contribution to the current depression of the elderly over the age of 65 [56]. Emotional intelligence was higher, especially in the elderly; therefore, it helps alleviate depression more quickly [56].

Furthermore, many studies have found an association between emotional intelligence and chronological age. A study conducted with 405 Americans aged 22–70 found a slightly significant positive relationship between emotional intelligence and age. The research found that older people had higher emotional intelligence than younger people. The findings reflected that emotional intelligence is the sum of the developmental skills acquired from the experiences encountered in life [57]. Kafetsios discovered age-related changes in emotional intelligence capacities. The emotional intelligence of older respondents was higher [58]. Similarly, many studies have found a positive relationship between age and emotional intelligence [59,60,61]. However, there have also been studies that did not show a significant relationship between age and emotional intelligence, albeit very few [62,63]. Many studies revealed the association between socioeconomic environment and emotional intelligence. Studies observed that those with low socioeconomic status had lower education, health, and psychological resilience due to high risks, and therefore lower emotional intelligence [64,65,66,67]. However, it was also seen that as socioeconomic status increases, individuals’ emotional intelligence decreases because they are less dependent and less motivated to identify their feelings of struggling with difficulties [68,69,70]. Studies have shown that low socioeconomic status reduces self-esteem, which gives individuals the ability to cope with problems [71]. As a result, these individuals may have low emotional intelligence by experiencing emotional instability in life [63]. According to the literature, the following hypotheses were proposed.

**Hypothesis** **1** **(H1).** *Emotional intelligence has a negative relationship with depression.*

**Hypothesis** **2** **(H2).** *Age and emotional intelligence have a positive relationship.*

**Hypothesis** **3** **(H3).** *SES and emotional intelligence have a positive relationship.*

### 2.3. Emotional Intelligence, Satisfaction with Life, and Depression as Mediator

Life satisfaction evaluates one’s life according to subjectively determined standards [72,73]. This concept is related to subjective well-being, which is defined as individuals’ emotional and cognitive evaluations of their lives [12,74,75]. Thus, life satisfaction is the state of taking pleasure from life, finding life meaningful, and evaluating people’s lives positively in accordance with their own criteria [75,76,77]. Life satisfaction differs from person to person. Factors such as health status, working life, economic level, psychological quality, education level, social environment, religion, spirituality, and perspective on the meaning of life affect satisfaction with life. People with high satisfaction with life may have the following attitudes [12,73,78,79]:

- Enjoying activities in their daily life;
- Having meaning and purpose in life, accepting the responsibilities of the past;
- Having the belief that they can achieve the goals;
- Accepting themselves as a valuable asset;
- Expecting to be optimistic about life.

Emotional traits are an important influence in enhancing persons’ cognitive evaluations of their own life satisfaction [80,81,82,83]. Studies conducted with university students examined the relationship between emotional intelligence levels and life satisfaction and found a positive and significant relationship between them [84,85]. In another study, emotional intelligence was positively associated with psychological well-being or life satisfaction measures and negatively with indicators of psychological disorders [36]. A negative relationship between life satisfaction and depression was also found [86,87]. Many studies determined that depression reduces satisfaction with life, and a negative relationship was revealed between them [77,86,88,89]. Moreover, the current literature has confirmed that the level of satisfaction with life decreased with the increase of depression of all segments of society, especially during extraordinary periods such as the COVID-19 pandemic [90,91,92].

**Hypothesis** **4** **(H4).** *Emotional intelligence has a positive effect on satisfaction with life.*

**Hypothesis** **5** **(H5).** *Depression has a negative effect on satisfaction with life.*

While emotional intelligence is used as an independent variable in many studies, it is used as a mediator and moderator variable in some [93,94,95,96]. A study conducted by Magnano et al. [97] revealed that individuals who better understand, access, and regulate their emotions are better at dealing with and adapting to challenging situations. According to some findings, the association between emotional intelligence and life satisfaction may be mediated by mental factors [26,98]. Moreover, due to the negative effect of emotional intelligence on psychological outcomes, stress and depression are used as mediators in many studies involving emotional intelligence [26,37,99,100]. Similarly, research revealed a significant role for depression as a mediator in the association between emotional intelligence and suicide risk in bullying victims [101]. Studies showed the favorable association of emotional intelligence with life satisfaction. Furthermore, a significant link to life satisfaction from emotional intelligence via stress by controlling demographics was found in surveys [37,102,103]. As a result, it is considered that emotional intelligence will lower the risk of depression and boost life satisfaction.

**Hypothesis** **6** **(H6).** *Depression has a mediating impact on the effect of emotional intelligence on satisfaction with life.*

### 2.4. Age and Family Type as Moderators

The relationship between satisfaction with life, depression, and age varies according to the studies. In some studies, there is no significant relationship between age and satisfaction with life and depression while there is a significant relationship in others. A person may have different expectations, activities, and challenges that may influence life satisfaction and depression in each period [75,77,104]. Therefore, it is possible to have different life satisfaction and depression in different life periods. Contrary to expectation, in a study conducted with 3287 people during the COVID-19 pandemic period, the depression levels of young people were significantly higher than the elderly due to not being able to go to school, increased career anxieties, and inability to socialize [84]. Although the pressure on young people increases during the COVID-19 pandemic, generally, young people are exposed to intense depression between the ages of 20–24, and depression decreases as age increases [105,106]. However, when health-related problems rise, depression increases significantly in the elderly. Depression may be caused by phenomenological differences between older and younger people and the link between age and psychomotor development [107]. Physical sickness, life experiences, lack of social support, and isolation are linked to depression, the most prevalent but treatable psychological disorder in senior years [108,109]. Adolescence is a developmental period that is marked by significant physical and psychological changes. Therefore, decline in life satisfaction during adolescence must be viewed as a developmental process [110]. In a study conducted with students, a negative correlation was found between age and satisfaction with life. However, no significant relationship was found in the sample in the same study, with no upper age limit obtained on the internet [111]. There are studies in the literature that find that satisfaction with life increases as age advances [112,113].

There may be differences between extended and nuclear family structures regarding family resources, parental support, communication opportunities between family members, use of financial resources, and socioeconomic status [114]. Extended families are expected to have less material and more interaction opportunities, whereas the opposite is expected in nuclear families. The number and quality of contacts between family members can have an impact on their personalities and psychologies. Individuals adapt their own emotions and behaviors in line with their social environment, parents’ expectations, and the quality of their emotionality, attitudes, and relationships with their parents [115]. The extent and nature of the interactions between family members may affect individuals’ characters and psychologies. Therefore, emotional intelligence, depression, and satisfaction with life may vary according to the family structures of individuals. A study conducted in Pakistan found that a suitable family environment with cohesiveness, supportive and caring family atmosphere, parents’ education levels, and children’s networks of friends positively correlated with emotional intelligence [115]. Studies found no difference in emotional intelligence between nuclear and extended family members [116,117]. Emotional intelligence was lower in broken, authoritarian, and neglectful families than in nuclear, extended, and democratic families [118]. In studies, fewer mental problems were discovered as the family grew. In this sense, mental health issues are mostly observed in single-parent families, then nuclear and lastly extended families [119]. In extended families with good parent and grandparent relationships and support, it was observed that family members have less depression and higher life satisfaction when their problems are shared [120,121,122,123].

There are studies with different results. Investigations revealed that intact and nuclear family members had fewer psychological symptoms than extended family members in Sweden [124,125]. Family size, life satisfaction, and depression may vary according to society. For example, those living in extended families may have preferred to live in a nuclear family if they migrated to other countries and married [126]. Other studies observed that life experiences and relationships in the family impact depression and the life satisfaction of individuals [127,128,129,130]. In a study conducted in Turkey, depression and life satisfaction of individuals did not differ according to extended and nuclear families [131]. A study in Pakistan found the depression of the elderly living in a nuclear family was almost twice that of those living in an extended family [132]. However, due to women’s workload in the extended family, women were more depressive than in nuclear families, whereas children in extended families had more positive values [133]. Thus, according to the studies, family type influences emotional intelligence, depression, and life satisfaction. Therefore, the following hypotheses were proposed.

**Hypothesis** **7** **(H7).** *Age has a moderating role in the effect of emotional intelligence and depression on satisfaction with life.*

**Hypothesis** **8** **(H8).** *Family type has a moderating role in the effect of emotional intelligence and depression on satisfaction with life.*

### 2.5. The Context of This Study

This study investigated the effect of adults’ emotional intelligence levels on depression and life satisfaction during the COVID-19 pandemic. Emotional intelligence is the ability to think about, organize, express, and accurately assess one’s own emotions, as well as the emotions of others. It is easy for people with high emotional intelligence to understand their own and others’ emotional states and manage difficult processes. Thus, individuals can use their emotional skills actively in their private and professional lives. In addition, emotional intelligence strengthens individuals’ resilience and ability to cope with rapid changes and problems, especially in today’s complex and depressive modern life. Thus, while individuals’ resistance against the problems they encounter in life increases, their satisfaction with life increases along with it.

**Figure 1:**
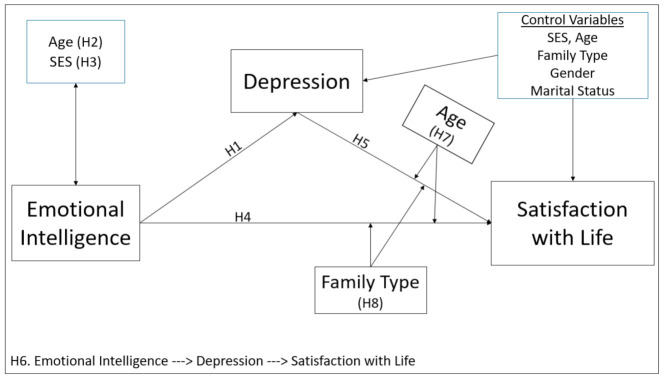
Schematization of the hypothesized model.

In the study, it was assumed that emotional intelligence increases satisfaction with life levels by reducing the depression of individuals. For this purpose, the conceptual model depicted in Figure 1 was designed. In the conceptual model, direct, indirect, and moderation analyzes were used. In addition, the *t*-test and ANOVA test were performed to determine the differences between groups. Finally, hypotheses for each relationship were defined and tested. Accordingly, emotional intelligence reduces depression, and decreased depression increases satisfaction with life. It was assumed that these relationships vary according to age level and family type.

## 3. Method

### 3.1. Procedure

Participants who supported the study voluntarily were reached through Survey Monkey, an online survey platform (https://tr.surveymonkey.com, accessed on 16 May 2021), between 10 February and 15 May 2021. The participants were informed about the purpose, method, and confidentiality of the collected data in research, and then their consent was obtained. Participants’ private information was not asked, their IPs were not kept, and their privacy was protected. Participants were allowed to start and end the survey or to withdraw from the survey at any time. It was determined that the survey was completed in 7 min on average. The survey was carried out according to the criteria of the Declaration of Helsinki. 

### 3.2. Measures

In order to determine the sociodemographic status of the participants through the personal information form, questions asked about gender, age, marital status, family type, education levels of the parents, and income status of the family. To discover the participants’ socioeconomic status, the parents’ educational status, and the family’s income levels, which were designed in five categories, were computed and averaged. A new factor called socioeconomic status (SES) was generated. The SES factor was used in the model with other control variables.

The Trait Emotional Intelligence Questionnaire–Short Form (TEIQue-SF) was developed by Petrides and Furnham [134,135], and later it was adapted into Turkish by Deniz et al. in 2013 [84]. In this study, the Turkish version was used. In the Turkish version, two different samples were used, and both of them were found to have high goodness of fit values. Internal consistency values for each sample were 0.81 and 0.86. The Turkish version of the Trait Emotional Intelligence Questionnaire–Short Form consists of 20 items, and a seven-point Likert-type scale with response options between strongly disagree (1) and strongly agree (7) was used. The higher the score obtained from the scale, the higher the emotional intelligence. The internal consistency value of the current study was α = 0.85. 

Satisfaction with life scale (SwL) was developed by Diener et al. in 1985 [136], and later adapted into Turkish by Dağlı and Baysal in 2016 [12]. The scale consists of five items to determine satisfaction with life scale, and a five-point Likert type scale was used in the answers, ranging between strongly disagree (1) and strongly agree (5). The internal consistency of Turkish adaptation was α = 0.88. Higher scores mean higher life satisfaction. The internal consistency ratio of the current study was α = 0.85.

Lovibond and Lovibond developed the Depression Anxiety Stress Scale (DASS-21) in 1995 [137]. The scale has 42 items in total, and a 4-point Likert-type scale is used for the items. With the studies carried out by Henry and Crawford in 2005 [138], the scale was reduced to 21 items as a short form. The Turkish validity and reliability study of the short form of the scale was carried out by Yılmaz (2017) [38]. There are seven questions in each scale to measure the dimensions of depression, stress, and anxiety. A four-point Likert-type scale was used with answers between not suitable for me (0) and completely suitable for me (3). Only the seven-item depression subscale was used for this study. A high score on the scale means high depression. The internal consistency value of the current study was α = 0.883.

### 3.3. Statistical Analyses

This study used a descriptive and quantitative method in conjunction with a cross-sectional design on a convenience sample described by gathering related data at a specific point in time [139]. After the survey was completed on Survey Monkey in the online environment, the data were cleaned and organized in the MS Excel program (Microsoft, Albuquerque, NM, USA). Then, the data edited with MS Excel were transferred to IBM SPSS statistical software (IBM, Armonk, NY, USA) [140], and the reverse coded questions were recoded to be analyzed. Afterward, confirmatory factor analysis (CFA) was used to see the suitability of the proposed measurement model using Amos 25 (IBM, Armonk, NY, USA) [141] and to ensure the construct validity of the factors. After reaching the values required for the goodness of fit of the measurement model, the SPSS data file, in which scales were generated by data imputation in the Amos program [141], was created. Finally, frequency, correlation, multiple direct regression, mediation, and moderation analyzes were conducted on the scaled SPSS data set, respectively. PROCESS-Macro Plug-in in SPSS (The Guilford Press, Calgary, AB, Canada) was used for mediation and moderation analyses [142]. To graph moderation results for two-way interactions, a simple slope test was performed [143]. PROCESS-Macro Model 15 was used to test the model. The statistical power of a study ranges from 0.00 to 1.00. As a result of the power analysis, the value should be 0.80 and above [144]. With the analysis performed using Gpower software (Universitat Kiel, Kiel, Germany) [145], the value of this study was found to be 0.95. The level of significance criteria in the research was at 0.05.

### 3.4. Confirmatory Factor Analysis

Confirmatory factor analysis (CFA) was used for factor analysis of the scales used in the study. The construct validity of the components was assessed using CFA, a sound method for studying latent component effects that directly analyzes assumptions a priori on relationships identified between variables [146]. A three-factor model was created with the scales used in the study. CMIN/DF, IFI, CFI, NFI, GFI, and RMSEA measurement values were used for goodness of fit values. In the criteria of the measurement values, the cut-off values determined by Kline were taken as a basis [147].

The measurement results were determined to be within the cut-off levels in the first test of the measurement model using confirmatory factor analysis (CMIN/DF = 3.105; RMSEA = 0.051; GFI = 0.911; CFI = 0.903; TLI = 0.907; IFI = 0.922; NFI = 0.898). When the modification indices were studied, it was discovered that generating covariances between the four high covariances would improve the measurement values. The final test results were determined to fit well with the research conceptual model as a result of the additional modifications, and the values were within high fit values (CMIN/DF = 2.269; RMSEA = 0.047; GFI = 0.927; CFI = 0.942; TLI = 0.933; IFI = 0.942; NFI = 0.901), as listed in Table 1.

## 4. Findings and Hypotheses Tests

### 4.1. Descriptive Statistics

Participants were a total of 578 adults over the age of 18 residing in Istanbul. Most of the questionnaires were answered and distributed by the Faculty of Health Sciences students, who are mostly women. In addition, the majority of the respondents of the questionnaires distributed by these students were women and youth. All of the participants were included in the study because they answered all the questions. Most of the participants were women (78.5% female, 21.5% male), the majority of them were young, so the average age was relatively low (Mean = 25.74, SD = 8.326), and most of them were single (23.5% married, 76.5% single). Furthermore, it was found that the family type of the majority of the participants was small (small family 89.3%, extended 10.7%), and their socioeconomic status (SES) was moderate (Mean = 2.497, SD = 0.888), as listed in Table 2.

### 4.2. Means, Standard Deviations, and Correlation Analyses

Table 3 shows the correlation, mean, and standard deviation values of the variables. According to this table, a positive correlation was found between the age variable and emotional intelligence (r = 0.125, *p* < 0.01) and a negative correlation with depression (r = −0.154, *p* < 0.01). A positive association was observed between socioeconomic status and emotional intelligence (r = 0.118, *p* < 0.01), and satisfaction with life (r = 0.155, *p* < 0.01). It was understood that there is a strong positive relationship between emotional intelligence and satisfaction with life (r = 0.623, *p* < 0.01) and a strong negative relationship between depression (r = −0.753, *p* < 0.01). A negative correlation was revealed between satisfaction with life and depression (r = −0.642, *p* < 0.01). According to the results, hypotheses H1, H2, and H3 were accepted. 

### 4.3. Direct and Interaction Effects

Table 4 shows the effects of main and interaction variables on dependent variables. In Step 1, it was understood that the effect of EI on depression is negative (B = −0.392, *p* < 0.001), while gender and marital status were positive (B = 0.756, B = 0.091, *p* < 0.05, respectively). In Step 2, it was found that EI had a positive effect (B = 0.212, *p* < 0.001), depression had a negative effect (B = −0.512, *p* < 0.001), SES had a positive effect (B = 0.076, *p* < 0.001), and age and gender had a negative effect (B = −0.007, B = −0.196, *p* < 0.05, *p* < 0.001, respectively) on satisfaction with life. In Interactions 1 and 2, moderator effects of age and family type variables were tested. In Interaction 1, the interaction of the moderation variable age with EI (B = −0.008, *p* < 0.05) and DEP (B = −0.021, *p* < 0.05) had a significant effect on SwL. In Interaction 2, the interaction of moderation variable FT with EI did not significantly affect SwL (B = 0.015, *p* > 0.05), whereas FT’s interaction with DEP on SwL (B = 0.050. *p* < 0.05) was significant. Therefore, hypotheses H4 and H5 were accepted.

### 4.4. Indirect Effects

In order to reach the results of the mediation analysis in the study, firstly, direct regression analyzes were performed between the variables. As seen in Table 4 and Step 1–2, emotional intelligence significantly affected depression, and depression significantly affected satisfaction with life. Therefore, for the mediation analysis, a significant relationship was established between the independent and mediating variables and between the mediating and dependent variables. In other words, it was observed that a significant relationship continued through depression, which was the mediating variable from emotional intelligence to satisfaction with life. Then, indirect analysis was performed to detect the mediation effect. In Table 5, the results of the indirect analysis are given. Accordingly, the mediation effect of emotional intelligence on satisfaction with life was determined through depression (γ = −0.2005, SE = 0.0267, 95% CI [0.1488, 0.2529]). Therefore, hypothesis H6 was accepted.

### 4.5. Moderation Effects

**Figure 2:**
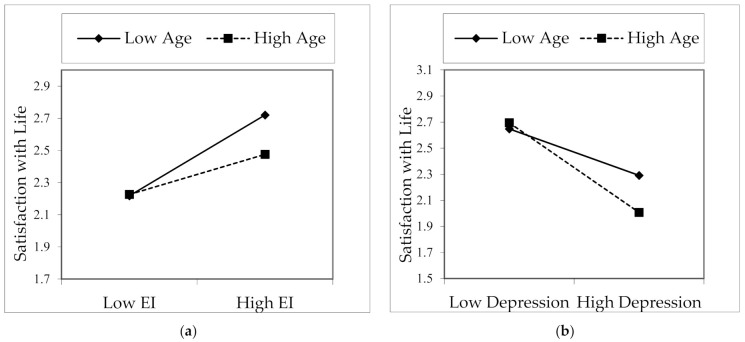
Interaction effect of age and EI (**a**) and age and depression (**b**) on satisfaction with life.

In the study, the moderation effect of age and family type in the effect of emotional intelligence and depression on satisfaction with life was included in the conceptual model, shown as in Figure 1. The results of the moderation analyses were shown in Interactions 1 and 2, in columns in Table 4. In Interaction 1, the effect of the interaction variable (EI × Age) between the age variable and EI on satisfaction with life was significant (B = −0.008, *p* < 0.05). Accordingly, the effect of emotional intelligence on satisfaction with life was different between those who are one standard deviation below the mean age (low age) and those who are one standard deviation above the mean age (high age). As illustrated in Figure 2a, satisfaction with life levels was higher in lower-aged individuals than higher-aged as emotional intelligence increases. In other words, the increase in the emotional intelligence of those with a low age leads to an increase in their satisfaction with life more than those who are older. 

The effect of the interaction between age and depression (DEP × Age) on satisfaction with life was also significant (B = −0.021, *p* < 0.05). The impact of depression on satisfaction with life was different between those who are one standard deviation below the mean age (low age) and those who are one standard deviation above (high age). As shown in Figure 2b, it was understood that the increase in depression generally decreased satisfaction with life, but the decrease in satisfaction with life was higher in those with higher age compared to those with lower age. Therefore, it is understood that the negative effect of depression affects satisfaction with life more adversely among older people. In the light of the results, hypothesis H7 was accepted.

**Figure 3:**
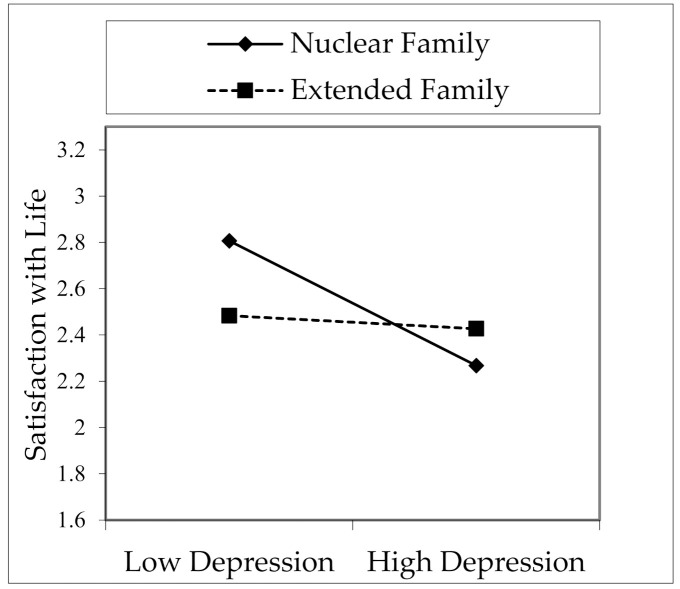
Interaction effect of family type and depression on satisfaction with life.

Table 4 and Interaction 2 show the interaction effects of family type moderation variable and emotional intelligence and depression on satisfaction with life. The impact of family type and emotional intelligence interaction variable (EI × FT) on satisfaction with life was not significant (B = 0.015, *p* > 0.05). However, the effect of the interaction variable (DEP × FT) between family type and depression on satisfaction with life was found to be significant (B = 0.050. *p* < 0.05). In this sense, the effect of depression on satisfaction with life was negative. However, this effect was more negative in nuclear families than in extended families. In other words, it was revealed that the negative impact of depression on satisfaction with life is much less in extended families, as seen in Figure 3. Therefore, hypothesis H8 was partly accepted.

### 4.6. The Results of Proposed Research Model and Hypotheses

**Figure 4:**
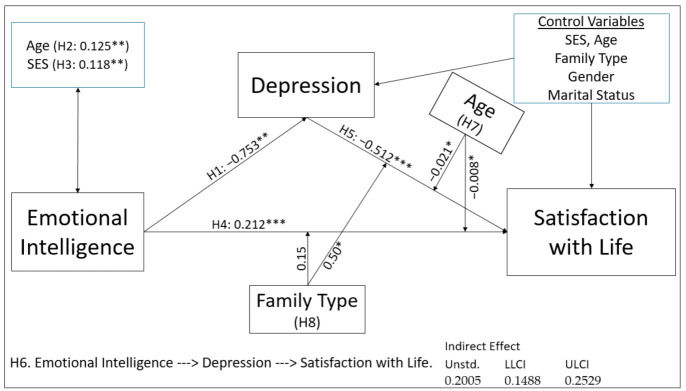
The results of proposed research model, * *p* < 0.05, ** *p* < 0.01, *** *p* < 0.001.

Correlation, direct, indirect, and moderation analyzes were performed with the variables in Figure 1, where the conceptual model of the study was shown, and then the results obtained were shown in Figure 4. The results were written next to the hypotheses or on each arrow line, with both the hypotheses and the results of the hypotheses and their significance values. The indirect hypothesis (H6) was written at the bottom of Figure 4, and the significance values were shown next to the hypothesis. A significant mediating effect of depression was found in the effect of emotional intelligence on satisfaction with life, as seen in Figure 4. In addition, there was a moderation effect with age and family type.

The direct, indirect, and moderation effects shown in Figure 2 were tested. Obtained coefficients and significance levels are placed in Figure 4. The results obtained as a result of the tests of the hypotheses are listed in Table 6 below. According to the study results, while all the hypotheses between H1–H7 were fully supported, only H8 was partially supported. In the discussion part, all hypotheses and research questions were evaluated in detail.

## 5. Discussion

In today’s societies, the nature of both individual and social relations is changing as life becomes increasingly complex. Social and economic problems that have emerged for several decades are rapidly affecting individuals at a global level. It is understood that the problems that arise with the COVID-19 pandemic negatively affect individuals’ physical and psychological states [90]. This study focused on the effect of emotional intelligence, which improves the ability of individuals to cope with psychological problems that arise as a result of the difficulties they encounter. Emotional intelligence is defined as comprehending, assessing, controlling, and regulating emotions [8,9,148]. Understanding and managing feelings also improve problem-solving abilities, the capacity to control negative emotions, and reduce mental effects such as depression. In this respect, the protective role of emotional intelligence is important in relieving increasing individual concerns and psychological problems during the COVID-19 period [14,15,32,149].

The current study examined the mediation effect of depression in the impact of emotional intelligence on satisfaction with life and the moderation effects of age and family type. Age, gender, socioeconomic status, marital status, and family types of individuals were used as control variables. As a result of the correlation, regression, mediation, and moderation analyses, the hypotheses between H1–H7 were fully supported, while the H8 hypothesis was partially supported. Based on the literature and the results of the hypotheses, direct, mediation, and moderation analyses and the research question asked in the introduction part were evaluated below.

### 5.1. Direct Effects

Significant associations between emotional intelligence and depression, socioeconomic status, and age were revealed in this study, and hypotheses H1, H2, and H3 were supported. According to the result of the H1 hypothesis, it was understood that depression decreased as emotional intelligence increased. Since both emotion and intelligence are active in mental processes, emotional intelligence has a protective and preventive effect on mental problems [22]. The result that emotional intelligence, an effective factor in coping with problems, has a negative effect on depression seems to be compatible with the findings obtained from the literature [33,36,37,150]. 

It was understood that emotional intelligence, which gives individuals the ability to understand, control and manage their own and others’ emotions during extraordinary times such as disasters and pandemics, increases as age (H1) and socioeconomic status (H2) increase. As the age increases, the rise in responsibilities and experiences of individuals contributes to developing their emotional intelligence. In the literature, it was observed that emotional intelligence increased with rising age [57,58,59,60], but some studies could not find any relationship [62,63]. In this study, a positive correlation was found between socioeconomic status and emotional intelligence. However, it was understood that there are two different views on this issue in the literature. On the one hand, it is emphasized that since SES will increase the education, health, and psychological resilience of individuals, they will have more opportunities and, therefore, high emotional intelligence [64,65,66,67,151]. On the other hand, as individuals’ SES increases, individuals’ emotional intelligence declines because they become less dependent and driven to identify their thoughts of being stuck in a challenging situation [68,69,70,71]. Individuals who have education, health, communication skills, and a good socioeconomic level that strengthens them will subsequently increase their emotional intelligence. The increase in the possibilities and opportunities for individuals to have these characteristics at older ages will also contribute to the increase of their emotional intelligence.

The study supported H4 and H5 hypotheses since emotional intelligence had a positive (H4) and depression (H5) negative effect on satisfaction with life. Emotional intelligence positively affects satisfaction with life levels as it improves individuals’ relationships and communication skills and increases their ability to cope with problems. Individuals who can control their own and others’ emotions will have more empathy, encounter fewer problems, and ultimately reach more satisfaction with life. The positive relationship between emotional intelligence and satisfaction with life revealed in the study is supported by current literature [80.81,84,102]. Depression has a characteristic cluster that can vary from ordinary sadness to severe psychotic symptoms. In this study, depression decreased life satisfaction, which is consistent with the existing literature [86,87,88,89,90]. Depression can be caused by various social, economic, emotional, and biological factors, negatively affecting satisfaction with life. It has been observed that people’s stress, anxiety, and depression increase in unusual times such as COVID-19 and disasters [90]. In these extraordinary times, people’s fears of uncertainty, future and career anxieties, fear of losing their jobs, and the risk of losing their health, all increase, thereby negatively affecting their psychological well being.

### 5.2. Mediation Effects

The study revealed that emotional intelligence, depression, SES, age, and gender effectively predicted satisfaction with life. Moreover, hypothesis 6 (H6) was supported since it was determined in the indirect analysis that depression had a mediating effect on the effect of emotional intelligence on satisfaction with life. Therefore, the significant effectiveness of depression as a mediator variable was found in the relationship between emotional intelligence, the dependent variable, and satisfaction with life, which is the independent variable. When depression was included as a mediator variable, the total effect was reduced by almost half from B = 0.4128 to B = 0.2123 as the direct effect. Thus, with the inclusion of the depression mediator variable in the model, the partial mediation effect of depression was realized as the direct effect between emotional intelligence and satisfaction with life was less than the total effect, and the significance status was still maintained.

In the literature, significant mediation effects of psychological outcomes such as depression, stress, and anxiety have been observed in the relationship between emotional intelligence and satisfaction with life [37,102,103]. Depression is one of the most common mental illnesses in the modern era. Problems such as career, unemployment, poverty, health, education, and family that individuals encounter even in ordinary periods lead to the rise of depression. Moreover, in extraordinary situations such as the COVID-19 pandemic, depression has affected all segments of society, along with stress and anxiety [90]. Therefore, as can be seen from the mediation analysis, it accounted for almost half of the total effect between emotional intelligence and satisfaction with life due to the widespread impact of depression during the COVID-19 period. If this finding is evaluated together with the result that emotional intelligence strongly reduces depression, the importance of emotional intelligence becomes more remarkable. Therefore, it is important to develop emotional intelligence in individuals from childhood to better struggle with extraordinary situations such as the COVID-19 pandemic.

### 5.3. Moderation Effects

Moderation analyses were conducted to understand whether the effects of both emotional intelligence and depression on satisfaction with life differ according to age and family type. Moderation analysis results using SPSS Process Macro Model 15 were shown in the interaction 1–2 columns in Table 4. Thus, hypothesis 7 (H7) was supported since it was determined that the age variable had a significant moderation effect in the effect of emotional intelligence and depression on satisfaction with life. According to this, those with a lower age have higher levels of emotional intelligence, which leads to higher levels of life satisfaction than those who are older. Furthermore, an increase in depression lowered overall satisfaction with life, but the decline in satisfaction with life was more considerable in older than younger. In the literature, different results were found between age and depression in different periods. Especially during the COVID-19 period, young people have higher levels of depression compared to the elderly due to problems such as career, employment, education, and inability to socialize [77,90]. However, it has been seen that depression increases in the elderly when they are exposed to physical problems, lack of social support, and exclusion [108,109]. Studies generally have found a positive relationship between emotional intelligence and age. Many studies have found a positive relationship between age and emotional intelligence [59,60,61]. However, a few studies did not find a significant association between age and emotional intelligence [62,63]. It was found that as the age variable increased, emotional intelligence increased. Thus, satisfaction with life also improved because, according to the findings, emotional intelligence is the sum of the developmental skills acquired or learned in life events.

Another moderation analysis was conducted with family type. There was no moderation effect of family type in the impact of emotional intelligence on satisfaction with life. However, it was understood that the effect of emotional intelligence on satisfaction with life was positive, and the extended family had a more impact on this effect. Although this result was not statistically significant, it coincides with the expectation that emotional intelligence is more functional in extended families and will increase individuals’ life satisfaction more than nuclear families [114,115,152]. A significant moderation effect was found in the effect of depression on satisfaction with life. Accordingly, depression had a negative effect on life satisfaction. This influence was, however, more negative in nuclear families than in extended families. Namely, it was discovered that the negative effect of depression on life satisfaction is much lower in extended families than in nuclear. Although there are no differences between nuclear and extended families in some studies, many have found differences among family types, depression, and satisfaction with life. There may be differences between extended and nuclear families due to parental support, interaction and socialization opportunities, and socioeconomic status [114]. While more interaction, knowledge and tradition transfer, and economic prospects are expected in extended families [115], the opposite is expected in nuclear families. Individuals unconsciously adopt the attitudes they interact with the social environment, and they become more harmonious with the social environment over time. Opportunities offered by extended families to individuals such as bonding with the past, the tendency to understand and solve problems within the family, and allocating more time to individuals reduce their depression [119,120,121,122,123]. It was understood that emotional intelligence decreased satisfaction with life by reducing depression and the effect of depression was less in extended families. However, it was seen that nuclear family members may face more mental problems due to more burdens, insufficient social environment, and low interactions [133]. In the extended family, family members are generally in solidarity with each other. As the number of individuals in the family increases, the number of good and bad cases observed increases. When individuals have reliable family members with whom they can share their problems, they can get the support they need to resist troubles and produce solutions. Therefore, it can be easy to solve the issues that may cause serious mental issues by sharing the problems and burdens within the family quickly. Since the extended family will consist of people with different personality traits, it will increase individuals’ communication skills, the ability to share tasks, and teamwork skills. Therefore, it will improve their emotional intelligence and prepare for professional life.

### 5.4. The Evaluation of Research Question

Before starting the study, a research question was asked: How do emotional intelligence and sociodemographic factors have a role in increasing satisfaction with life? For this purpose, research was conducted on to what extent and how the variables of emotional intelligence, depression, socioeconomic status, age, gender, marital status, and family status predict satisfaction with life. Then, after illustrating the conceptual model in accordance with the literature, hypotheses were designed for each relationship and effect that will lead to the result. Finally, the research question was evaluated according to the hypotheses’ findings found as a result of the tests and the current literature. As a result, it was determined that emotional intelligence, depression, socioeconomic status, age, and gender predicted satisfaction with life significantly. In addition, it was found that depression had a significant mediating role in the relationship between emotional intelligence and satisfaction with life. Furthermore, the significant moderating role of age and family type was revealed. In addition, in the ad hoc causal chain serial mediating analysis, it was understood that the family’s socioeconomic status increased emotional intelligence, increased emotional intelligence decreased depression, and decreased depression affected satisfaction with life less negatively.

During the COVID-19 pandemic, individuals face severe difficulties in both individual and social areas due to education, health, career, and employment. In addition, the pandemic forces every field at a global level to an unusually online environment causing tensions in individuals. Therefore, emotional intelligence, which brings communication and relationships to a reasonable and manageable level, should be strengthened in individuals, especially from childhood, to increase the life satisfaction of individuals in difficult times such as the pandemic and to make them more able to cope with difficulties such as stress, anxiety, and depression caused by challenges. Emotional intelligence, which contributes positively to a more intelligent, more determined, and meaningful life of individuals, also contributes to empathy with its ability to understand, control, and manage emotions. Therefore, recognizing emotions from childhood, displaying empathetic behaviors, talking about emotions abundantly, using interactive communication language, giving responsibilities according to abilities, making them gain artistic skills appropriate to their interests, and problem-solving skills with physical and mental activities, will improve individuals’ emotional intelligence.

## 6. Study Limitations

This study investigated the effect of emotional intelligence on life satisfaction, the mediating role of depression in this relationship, and the moderating effects of age and family type. Since the method of this research was cross-sectional, this should be considered when interpreting the results. The study cannot be generalized to Turkish society or other societies because it was conducted with limited participants. Since the study was conducted within the COVID-19 pandemic process and conditions, the results are valid for the COVID-19 period. In addition, the fact that elderly, men, married people, and extended family members are in lower numbers than other groups is an essential limitation of this study. Since the research was conducted only online and quantitatively, the participants’ thoughts, attitudes, and one-to-one emotional reactions could not be understood. In addition, since only the trait dimension of emotional intelligence was used, other dimensions could not be evaluated.

## 7. Conclusions and Some Implications

It is an essential trait that individuals can understand, control and manage their own and others’ emotions in unusual times like the COVID-19 pandemic. Emotional intelligence, which will bring this feature to individuals, has a positive effect on satisfaction with life as well as its protective and therapeutic ability against mental problems. This study determined that depression has a mediating role in the positive impact of emotional intelligence on satisfaction with life, especially during the COVID-19 pandemic. Furthermore, the age variable had a full and family type variable had a partial moderating effect. In addition, the family’s socioeconomic status was used as an independent variable, and it was found to increase the emotional intelligence of individuals. Moreover, a positive relationship between the age variable and emotional intelligence can be explained by the responsibilities and experiences gained with increasing age, which improves the emotional intelligence of individuals. Furthermore, the ad hoc serial mediation analysis based on a causal chain revealed that the family’s socioeconomic level boosted emotional intelligence, then increased emotional intelligence lowered depression and, lastly, reduced depression had a less negative effect on satisfaction with life. Therefore, it is necessary to consider and implement policies and practices that will support the development of emotional intelligence.

Emotional intelligence should be gained from childhood, especially in the family, neighborhood, social environment, and school. Thus, individuals will gain the ability to cope with adolescence and adulthood problems more effectively. For example, parents should use interactive language and show empathetic approaches to recognize their children’s emotions and improve their communication skills. In addition, the family and the school should consider activities and artistic activities that will develop the sense of responsibility and abilities of children and young people. Ultimately, mental and physical activities should be brought to the agenda at family, civil society, and school levels to support problem-solving skills and strengthen individuals’ emotions and moods by policymakers.

## Figures and Tables

**Table 1:** Confirmatory factor analysis values.

Criteria	Normal Values	Strong Values	Present Values
CMIN/DF (x^2^/df)	<5	<3	2.889
RMSEA	<0.08	<0.05	0.036
GFI	>0.90	>0.95	0.976
CFI	>0.90	>0.95	0.983
TLI	>0.90	>0.95	0.983
IFI	>0.90	>0.95	0.974
NFI	>0.90	>0.95	0.983

*Notes*. CMIN/DF = Chi-square Fit Statistics/Degree of Freedom, RMSEA = Root Mean Square Error of Approximation, GFI = Goodness-of-Fit Index, CFI = Comparative Fit Index, TLI = Tucker-Lewis Index, IFI = Incremental Fix Index, NFI = Normed Fit Index.

**Table 2:** Descriptive statistics.

Variable	Category	*f*	%	M	SD
Gender					
	Female	454	78.5		
	Male	124	21.5		
Age				25.74	8.326
Marriage					
	Married	136	23.5		
	Single	442	76.5		
Family Type					
	Small	516	89.3		
	Extended	62	10.7		
SES				2.497	0.888
Total		578	100%		

*Notes.* M = Mean, SD = Standard Deviation, SES = Socioeconomic Status.

**Table 3:** Correlation, mean, and standard deviation analyses.

No.	Variables	Mean	Sd.	1	2	3	4
1	Age	25.74	8.33	1			
2	SES	2.50	0.89	−0.028	1		
3	EI	3.46	0.93	0.125 **	0.118 **	1	
4	SwL	2.18	0.62	0.033	0.155 **	0.623 **	1
5	DEP	0.79	0.48	−0.154 **	−0.041	−0.753 **	−0.642 **

*Notes*. EI = Emotional Intelligence; Dep = Depression; SWL = Satisfaction with Life; SES = Socioeconomic Status; ** *p* < 0.01.

**Table 4:** Main and interaction effects on DEP and SwL.

Variables	Step 1: DEP			Step 2: SwL			Interaction 1: SwL			Interaction 2: SwL		
	B	SE	*p*	B	SE	*p*	B	SE	*p*	B	SE	*p*
(Constant)	1.935	0.266	<0.001	2.314	0.237	<0.001	2.42	0.145	<0.001	2.63	0.20	<0.001
EI	−0.392	0.014	<0.001	0.212	0.031	<0.001	0.203	0.031	<0.001	0.05	0.12	0.641
DEP				−0.512	0.059	<0.001	−0.541	0.06	<0.001	−1.06	0.23	<0.001
EI × Age							−0.008	0.004	0.044			
DEP × Age							−0.021	0.008	0.013			
EI × FT										0.15	0.10	0.155
DEP × FT										0.50	0.20	0.014
*Control Variables*												
SES	0.225	0.015	0.132	0.076	0.021	<0.001	0.075	0.021	<0.001	0.08	0.02	<0.001
Age	−0.001	0.002	0.753	−0.007	0.003	0.019	−0.007	0.003	0.02	−0.01	0.01	0.015
Gen. (1–2)	0.756	0.032	0.019	−0.196	0.046	0.000	−0.187	0.046	<0.001	−0.19	0.05	<0.001
Mar. (1–2)	0.091	0.042	0.030	−0.066	0.06	0.270	−0.056	0.06	0.352	−0.06	0.06	0.277
FT (1–2)	−0.073	0.043	0.087	−0.099	0.06	0.103	−0.092	0.06	0.127	−0.08	0.06	0.186
F		133.24			78.87			62.48			62.60	
*p*		<0.001			<0.001			<0.001			<0.001	
R2		0.583			0.492			0.498			0.498	

*Notes*. EI = Emotional Intelligence; DEP = Depression; SwL = Satisfaction with Life; SES = Socioeconomic Status; Gen = Gender 1: Female, 2: Male; Mar = Marriage, 1: Married, 2: Single; FT = Family Type, 1: Small, 2: Large.

**Table 5:** Total, Direct and Indirect Effects of Emotional Intelligence (EI) on SwL.

Total Effects of EI on SwL					Unstand.	SE	LLCI	ULCI
					0.4128	0.0216	0.3703	0.4553
Direct Effects of EI on SwL								
					0.2123	0.0309	0.1516	0.273
Indirect Effects of EI on SwL via DEP								
Independent		Mediator		Dependent	Unstand.	SE	LLCI	ULCI
EI	>	DEP	>	SWL	0.2005	0.0267	0.1488	0.2529

*Notes.* EI = Emotional Intelligence, DEP = Depression, SWL = Satisfaction with Life, LLCI = Lower Level of Confidence Interval, ULCI = Upper Level of Confidence Interval.

**Table 6:** Summary of hypotheses testing results.

No.	Relationship	Proposed Relationship	Support
H1	EI–DEP	Negative	Yes
H2	Age–EI	Positive	Yes
H3	SES–EI	Positive	Yes
H4	EI–SwL	Positive	Yes
H5	DEP–SwL	Negative	Yes
H6	EI–SwL	Mediated by Depression	Yes
H7	EI and DEP–SwL	Moderated by Age	Yes
H8	EI and DEP–SwL	Moderated by Family Type	Yes (Partly)

## Data Availability

The data presented in this study are available on request from the corresponding author.
